# The ‘Voice’ of Key Stakeholders in a School Food and Drink Intervention in Two Secondary Schools in NE England: Findings from a Feasibility Study

**DOI:** 10.3390/nu11112746

**Published:** 2019-11-12

**Authors:** Lorraine McSweeney, Jen Bradley, Ashley J. Adamson, Suzanne Spence

**Affiliations:** 1 Human Nutrition Research Centre, Population Health Sciences Institute, Faculty of Medical Sciences, Newcastle University, Framlington Place, Newcastle upon Tyne NE2 4HH, UK; lorraine.mcsweeney@newcastle.ac.uk (L.M.);; 2 Fuse, UKCRC Centre for Translational Research in Public Health, UK

**Keywords:** school food, pupils, food choice

## Abstract

**Background**: Overweight/obesity affects one-third of UK 11–15-year olds. Individually focussed interventions alone have limited effectiveness. Food choice architecture approaches increase the visibility and convenience of foods to facilitate the choice of ‘healthier’ foods and reduce ‘unhealthy’ foods. This qualitative component of a School Food Architecture (SFA) study aimed to determine the perceptions of pupils and staff in relation to school food provision and their perceptions of the intervention. **Methods**: Pupil focus groups and staff one-to-one interviews. Topic guides were developed from literature and in consultation with a Young Person’s Advisory Group. Thematic analysis was applied. Results: Focus group (*n* = 4) themes included: dining hall practices, determinants of choice, and aspects of health. Interview themes (*n* = 8) included: catering practices, health awareness, education, and knowledge of intervention. Pupils liked to purchase hand-held, quick to purchase foods potentially limiting the access to fruits and vegetables. Pupils were aware of ‘healthier’ food choices but would choose other options if available. **Conclusions**: Schools provide a daily school meal for large numbers of pupils, with time and dining environment constraints. Pupils consume 35–40% of their daily energy intake at school, therefore interventions enabling healthier eating in school are essential, including making healthier choices readily available and accessible.

## 1. Introduction

One-third of the 11–15-year olds in the UK are overweight or obese [1]. This has consequences for their emotional, behavioural and physical health and may result in morbidity and premature mortality in adulthood [2,3,4]. The UK government aims to halve childhood obesity rates by 2030 [5]. Chapter two of the Childhood Obesity plan sets out strategies to address this goal; including a commitment ‘to support all children with high quality nutrition’ [5]. Given the proportion of food that children eat at school [6], focusing on schools as an environment for promoting healthier food choices [5] has the potential to influence a substantial proportion of food intake across all social groups and offers the potential to form new food habits [7]. However, the reduction of overweight/obesity is a complex challenge. Major areas of concern are high intakes of energy, fat and sugar-sweetened beverages and low intake of fibre, fruit and vegetables [7]. The UK National Diet and Nutrition survey [8] reports that free sugars make up 14.1% of the 11–18-year olds daily calorie intake and sugar sweetened beverages amount for 22% of their diet. Fruit and vegetable consumption averages at 2.7 portions per day.

Encouraging healthy eating in children and young people is multifaceted and influenced by a myriad of external factors. Food-related decision-making processes are thought to be governed by a two-way system: the reflective system is driven by values and intentions; it requires thought [9]. The second, an automatic system requires little or no thought and is motivated by feelings and our environment [9].

The World Health Organisation (WHO) advocate population-based strategies which seek to change the social norm by encouraging a change in behaviour to improve health, however, behaviour change has proved challenging [10]. Current evidence suggests that changing the number of available food options or altering the positioning of foods could contribute to positive changes in behaviour. To enable more certain and generalisable conclusions about these potentially important effects, further research is warranted in real-world settings [11]. One approach to facilitate behaviour change is food choice architecture; that is increasing the visibility and convenience of foods to encourage the purchase and uptake of ‘healthier’ foods and reduce ‘unhealthy’ foods [12], and may reduce inequalities [13]. Reducing the cognitive demands of healthy food choice at the point of purchase has the potential to affect health behaviours [14]. 

This study aims to explore the perceptions of pupils, teaching and catering staff to school food provision, and the perceived impact of an intervention which repositioned drinks, cookies and fruit in the school dining hall.

## 2. Materials and Methods 

### 2.1. Study Design and Setting

Two secondary schools in North East England participated. The intervention focused on the placement of fruit, cakes/cookies and drinks. Fruit was placed in front of cakes/cookies and drinks were positioned according to sugar content, with water now positioned at eye level. Pupils participated in age-appropriate focus groups and school staff (teaching and catering) in one-to-one semi-structured interviews. Pupil focus-groups and staff interviews were conducted during the school day on school premises. Focus groups were facilitated by L.M. and S.S. and lasted approximately 1 h. Staff interviews were approximately 30 min and facilitated by L.M. 

### 2.2. Participants, Recruitment and Consent

Named contacts for the school were contacted by lead researcher (S.S.) by email or telephone to request help in organising the recruitment of pupils. A purposive sample [15] of pupils aged 11–16 years were provided with a study information sheet and invited to take part in one of the two focus groups (per school): ages 11–13 years and 14–16 years. Parent and pupil written consent was required prior to participating. 

A sample of teaching and catering staff were invited by email or telephone to take part in the one-to-one interview. Written consent was obtained before participation. 

### 2.3. Materials

The focus group and interview topic guides were developed from the literature and in consultation with the Young Person’s Advisory Group-North East (YPAG). The topic guides can be found in Appendix A and Appendix B. Participants were asked to discuss topics related to day-to-day practices of school food provision, satisfaction of school foods, perceptions of school regulations relating to school food provision, perceptions of health, and awareness, if any, of the intervention. 

### 2.4. Data Management and Statistical Analysis

Interviews and focus groups were digitally recorded with the participant’s consent and transcribed verbatim. Data was analysed using thematic analysis. NVivo software was used to aid indexing and charting [16]. Guided by the principles of grounded theory [17], the data was repeatedly read and coded independently by L.M. within a framework of a priori issues identified from the topic guide and by participants or which emerged from the data. Regular discussion and review of the analysis by L.M. and S.S. acted as a quality control measure. 

## 3. Results

### 3.1. Focus Groups

Eight pupils from each of the two schools (four in each age category; total *n* = 16) took part in an age separated focus group discussion. Three themes and 15 sub-themes were identified (Table 1). 

### 3.2. Dining Hall Day-to-Day Practices

Overall pupils were accepting of dining hall practices though there was some frustration over the length of queues and time they had to wait to be served. The necessity of having to queue often impacted on pupil’s knowledge of daily menus. Although many items such as sandwiches, pasta pots and pizza were available daily, pupils found it difficult to know what the hot meals/special offers would be:

*‘You can have a look. When you go in, there’s a big one (menu) on the side but sometimes it’s hard to track what day it is. When you actually get there (hot food counter), there is a piece of paper and it says what’s on today’s meal deals (FG4)’.*

Although the hot meal deals appeared to be popular among the pupils, as they represented good value, some pupils reported buying the items such as pizza or pasta pots daily for a speedy decision and purchase. Despite the popularity of the meal deals, some felt the choice was limited; every day the deal included a small drink and a cookie but an alternate type of dessert or fruit was classed as extra which not all pupils had the budget for:

*‘I think we should be allowed to get fruit and a drink because otherwise you’re only allowed to get your meal and fruit. I don’t think that’s right because I think people could be dying of thirst but they want to be really healthy and they don’t want to get a cookie. That means if you’ve got a drink, you would have to pay extra for it’. (FG4)*

Many pupils purchased food at the morning break, some would keep the item to eat at lunch-time thus removing the pressure to join the lunch-time queue. Items for sale at mid-morning included bacon rolls, sausage sandwiches and paninis. Pupils who ate a hot snack at break said that this often influenced what they might want to purchase at lunch-time:

*‘I just like taking a cookie and then a drink. Sometimes you don’t want a big meal. You just want something little to snack on’. (FG2)*

In one school the purchase of sweet items, such as the cookies, was limited to one a day, the other school did not appear to impose such limits. Staff reported they were not always able to monitor the pupil’s purchases because of the multiple till points in the dining hall and separate hall where sandwiches were sold. 

### 3.3. Aspects of ‘Healthy’ Eating

Pupils of all ages were aware of the importance of eating fruit for health, however, the availability, price and quality of the provided fruit was not always an incentive:

*‘I’m only allowed to spend £ 2.00 a day and like if I want a meal and some fruit I’ve got to pay extra’. (FG1)*

Whilst some of the younger pupils said they did not worry about their diet for health…

*‘Like with me being a younger kid I know what’s healthy and what’s not and I’ll admit I do eat unhealthy food, but yes, you can say, “Oh eat this instead of this,” but I’ll be honest, we won’t listen because we just think, “Oh we’re young, we can do whatever we want”’. (FG1)*

Pupils were aware of the disconnect between what they were being told and taught and what happened in reality:

*‘I think they have unhealthy foods on display. It’s like you’re telling us to be healthy but you’re showing us unhealthy food, of course we’re going to choose the unhealthy one instead of the healthy choice which you’re trying to make us choose. It’s pointless telling us to choose healthy stuff when you’re putting out unhealthy stuff for us to buy I think’. (FG2)*

One school regularly took part in charity fund-raising initiatives whereby staff and pupils were selling sweets and cakes:

*‘Yes, they sell anything that they can make money out of. So at the minute my form has just been selling ice pops because it’s been really hot. But some are selling donuts, cookies, chocolate, pick ‘n’ mix’. (FG3)*

Pupils were generally open for the school implementing strategies to encourage healthier choices and had some ideas:

*‘Lower the unhealthy stuff I think. Like, maybe not make it not every day. Instead of cookies one day have the fruit pots instead and then one day have the cookies have fruit pots so you’re like, “I might as well have something healthy since that’s all there is”’. (FG4)*

However, when asked if further restrictions should be made such as the blanket banning of certain foods there was strong opposition, especially in one school where ‘unhealthier’ items were not already restricted. Pupils said they would just go to the local shops or bring in items from home. Although pupils understood that fizzy drinks were considered unhealthy, the consumption of ‘juice’ (diluting juice) was thought to be an everyday necessity:

*‘I think you drink juice anyway in your normal day…I think it’s unfair if they went no juice because you’re drinking juice anyway so that’s not really going to make a difference. I think it’s more the food that you’re taking in’. (FG4)*

The pupils appeared satisfied with the choice of the current school drink provision and would not make any changes. Less acceptable was the current provision of free water. Although pupils in both schools reported the availability of water fountains there was reluctance to use them:

*‘It’s (water fountain) not like one of those ones where you put your cup underneath and press it, which I think would be such a good idea. There are other ones and it’s like a tiny little bowl about that big with a tap coming out of it and you press the button and it just trickles out of the top. But people put their mouth around it, so I’ve never got a drink out of there’. (FG3)*

### 3.4. School Food Architecture Intervention Implementation

The pupils were asked if they had noticed any changes to the dining hall in recent weeks. Pupils spoke of tables being moved around, walls being decorated and different cheese on the pasta. It was only with prompting that the pupils spoke about the introduction of the fruit pots:

*‘The fruit used to be put on a plate whereas now they make some effort with presentation, like all cut up and…they do little fruit pots as well’. (FG1)*

No other observations were made. 

### 3.5. Staff Interviews

One member of the teaching staff and three catering staff members were interviewed from each school (total *n* = 8). Eight themes and 25 sub-themes were identified (Table 2).

### 3.6. School Food Provision Is Important

There was an overwhelming consensus that providing pupils with ‘good’ food was vital. It was acknowledged that for certain pupils, a school meal may be the only hot meal they received that day:

*‘The school cares about the food here, they’re always trying to improve and do better—give better stuff and that—everything is fresh’. (ST03)*

Showing pupils a different side to food and diet was considered an important aspect:

*‘There is a different world of taste and nourishment and healthy eating, and we need to be showing them—you know, for some children who’ve never sat in a restaurant and eaten good quality restaurant food, that if we can be that representation in school as much as we can be, with different flavours, different cultural foods’. (ST01)*

### 3.7. School Catering Practices 

Interviewees spoke of the need to comply with the local council school food regulations. Menus were developed through development chefs. The importance of knowing what the pupils liked and would eat was stressed:

*‘I think you’ve got to know your kids and your kitchen and know what’s going to sell that day’. (ST02)*

Staff recognised that despite restrictions being put in place, for example, the amount of sugar that cakes, cookies and drinks were allowed to contain, it was not practical to enforce:

*‘However, where it falls down, is that, for example, you might see a homemade muffin, which has been made directly to the (regulated) recipe, but then a student can buy three of them. So there is no restriction on quantity. A student can buy, what a student wants’. (ST05)*

### 3.8. Types of Foods Pupils Buy

It was acknowledged that many pupils made use of the opportunity to buy hot snack items and sandwiches at break-time, this often impacted on their lunch-time choices:

*‘You have to be careful, because they might have had a bacon sandwich at break and then they want a biscuit at lunch. There’s still that bit about, “I can have three slabs of cake”. So I think there needs to be a bit more work done on volume and quantity of what they’re actually buying and do you introduce a healthier way of choices’. (ST05)*

The staff too were aware that many pupils would choose ‘easy’ options for speed and familiarity.

### 3.9. Day-to-Day Serving Practices and Issues

The complexity of dealing with the sheer volume of pupils at one lunch sitting was an everyday challenge:

*‘Yes, we’ve got a pasta area which is separate. We’ve got a hot service area, which is your main food, a chicken area, and a pizza area. It does take the pressure off the main hatch, but there is still a massive queue at the main hatch’. (ST03)*

*‘It’s pretty busy, because we haven’t got a massive dining hall. Obviously, we’re getting more and more kids. We only have one sitting at the moment. I think they’re getting rushed with their food. I think it’s like trying to get them in, fed, and out ready for more people coming in’. (ST04)*

The number of pupils being catered for made it impossible to monitor individual purchases, the school with purchasing restrictions in place suggested they found this difficult to enforce:

*‘I think it might just be in the school, you know, and that’s them saying no, one item, breakfast one item, one drink. And I have seen the children say, “I want two”. “No, you can’t, you’re only allowed one”. Send their friend back up or the other one, you know. Like sometimes you watch them and think, “You’ve just got that cookie for him”, but you can’t sort of stand there and argue and say, “No, that’s not for him”’. (ST02)*

### 3.10. Drinks and Food Considered to Be Less Healthy

One school which had ‘junk food’ restrictions tried to adhere to their policy:

*‘Yes, so we’ve obviously got food policy in place. We, on a general- around the corridors, at break and lunch, will take pop and anything like big packs of biscuits, big bags of sweets. We will go into assembly…just to remind- they’re non-negotiable, they’ll be taken off you or parents can come and collect them…we monitor it that way’. (ST01)*

Nonetheless, catering staff found the restrictions conflicting:

*‘That’s it, there is nothing stopping them going into the quad (separate school food purchasing area) and getting a cake and a cookie, and then coming back into the dining hall and saying, “I’ll have another cake, I’ll have a cookie and a flapjack”’. (ST03)*

### 3.11. Nutrition, Health Awareness and Education

The attitudes and pupil’s knowledge of diet were highlighted as being key:

*‘I think we are aware that being in a deprived area, there might be a number of students who haven’t eaten anything in the morning. There are also a lot of students, even if they could eat anything in the morning, they don’t and they don’t think that’s a problem. There is still the issue where they might think a packet of crisps is fine for breakfast’. (ST05)*

Providing dietary education to pupils of all ages was considered important:

*‘I think it would be better, almost if it was a curriculum requirement within PHSE (personal, social, health and economic: a school curriculum subject), in every year group, until they leave school’. (ST05)*

### 3.12. Perception and Knowledge of the School Foods Intervention

The catering staff were able to describe the changes that had been requested of them during the intervention and reported being happy to comply. There was some surprise expressed by the staff as the intervention had made them more aware of the drinks being sold and the high level of sugar contained in some:

*‘No, I think everything that they did was great because it opened my eyes, because of the sugar content, which I was so surprised at’. (ST02)*

The subtlety of the intervention was praised:

*‘I think the fact that it was subtly done, I think the children weren’t really- it was obviously subconscious decisions they were making, which I think is about product placement, isn’t it? And preparation for selling that type of thing’. (ST05)*

The introduction of the fruit pots sparked interest and extra consumption:

*‘The kids were asking, “Why are you getting all these pots?” Many of the kids did ask, “Oh, are you starting to do fruit up here. We never used to take fruit up to any other point, bar the main hatch. I think they noticed, “Oh, I’ll have fruit.” When it’s there, they were taking it’. (ST03)*

The increase in fruit purchases was thought to be due to the novelty factor:

*‘Sort of… Yes. Because it was amazing because they said, “How is it?” And I went, “Absolutely great.” They did 40 pots, you know, to start off with. Keep on the 40 pots, and then all of a sudden it was like 20 pots, and I’m saying, “Well, what’s happening? They’re not taking it, they’re (catering staff) just bringing it back”’. (ST02)*

There was some concern that the fruit pots would take longer to prepare, however, it was conceded that once in a routine the preparation was not onerous. 

There were fewer comments regarding the changes in the drink positioning although one school felt the quantities of water being sold had changed:

*‘I thought there was more water, because I’ll tell you why. I was doing the orders and I would get, say, six cases of water. I upped it to eight’. (ST02)*

### 3.13. How School Food Provision Could Be Improved or Enhanced

At the end of the interview staff were asked to describe their ideas or future plans to improve school food provision. 

*‘I’d suggested recently with student council that we give a reward system (for choosing healthier options) if we see good practice in place…… like through a prize at the end of term or something like that could be put into place for that’. (ST01)*

## 4. Discussion

Several barriers to pupils making healthier choices were identified. In both schools, pupils and staff spoke of the long queues and lack of time to sit and enjoy what was purchased; these issues have been reported in previous studies and negatively influence the eating experience [7]. There appeared to be an active intent by schools to provide more convenience hand-held type foods in several locations that would be quick to access and encourage pupils to leave the dining area quicker. Both schools sold hot sandwich type items at morning break which appeared to be popular. Some pupils stated that they would eat/keep these items as a lunch replacement and then purchase an additional snack/biscuit/drink at lunchtime. This practice potentially limits the pupil’s access to fruit, vegetables and salads. Also highlighted was the occurrence of individual pupils purchasing the same types of foods on daily basis such as pizza slices, paninis and pasta, again limiting the consumption of fruit and vegetables. Reasons for these habitual purchases were lack of awareness of the daily menu, speed of purchase and familiarity with prices. Regardless of the schools complying with the local council guidelines with regards to sugar content in recipes, the pupils’ capacity to purchase more than one ‘regulated’ item means they could exceed the daily recommendations. 

Pupils were aware of the health reasons behind the restrictions though and stated that if unhealthier items were available to buy then they would do so. It has been reported that children may be less able than adults to resist temptations in choosing unhealthier options if available [7]. It has been suggested that even adult study participants would be more likely to buy food items that are directly available to them as opposed to pictures or description of items [18]. Furthermore, it is established that having knowledge of ‘healthier/unhealthier’ foods alone is unlikely to influence pupil food choice [19]. Both staff and pupils felt there was too much availability of cookies/sweet items. Previous work with much younger pupils (kindergarten to 10 years) [20] suggests that the involvement of pupils in the design and promotion of healthy eating promotion materials may help to establish group norms about attitudes and preferences towards healthy food consumption. 

Despite pupils’ awareness of healthy eating guidelines there was opposition of an all-out ban of certain foods and drinks; especially for juice (diluting juice); juice was considered to be an essential component of the daily diet. Mâsse et al. (2014) [21] suggested that having access to sugar sweetened beverages (SSB) at school may disproportionately affect pupils from a less healthy home environment, as they are likely to consume SSB both at home and school. There were suggestions that if certain food items were removed, such as pizzas, pupils would just bring in packed-lunches or buy items from out-with school. There were examples of conflicting practices; one school regularly hosted charity fund-raising schemes by selling confectionery, cakes, etc. to pupils at discounted prices (cheaper than items sold in the dining room). This conflict did not go unnoticed with pupils commenting on the disconnect of healthy eating messages being promoted by the school and the availability of such foods. In older pupils, such as those in this study, perceived eating norms may act as a form of normative social influence whereby they may copy the behaviour of others when they are concerned with feeling socially accepted or establishing a relationship with the source of the influence [22].

With respect to the intervention, implementation did not seem to cause many difficulties for the staff. Some staff expressed surprise in the amount of sugar some of the drinks offered to pupils contained. The increased sale of the fruit pots was seen as a positive development by staff, despite a decline after two weeks. Pupils were not aware of the changes being made to the positioning of drinks and cookies, although with some prompting, they reported being aware of the introduction of the fruit pots. 

## 5. Limitations 

The data collected in this study were from two schools in the North East of England; the pupils who consented were selected purposively from the school council, therefore, opinions may not be representative of the general ‘school’ population or generalisable to other areas.

## 6. Conclusions

Secondary schools face many barriers and challenges in providing a school food service. As highlighted in the childhood obesity plan [5], the healthiest choice should be the easiest choice. However, as demonstrated in this study, the everyday practicalities of implementing this in a school setting is challenging. Pupils are receiving mixed messages in terms of education and the types and number of ‘unhealthy’ items they are able to buy, not just at lunch-time, but throughout the school day. Moreover, reducing unhealthier options but also giving pupils choice does not guarantee they will choose the healthier options [19]. As suggested by the pupils themselves, reducing the availability of ‘unhealthier’ options, by not selling cakes/cookies/pizza every day but limiting to once or twice a week, may be a more pragmatic approach. 

## Figures and Tables

**Table 1:** Themes and sub-themes from pupil focus groups.

Theme	Sub-Themes
Dining hall day-to-day practices	■ Perceptions of dining hall rules ■ Dining hall atmosphere ■ Having to wait in long queues ■ Knowing the daily menu and offers ■ Use of meal deals ■ Pre-ordering of food items ■ Pupils can buy snack foods ■ Some food items sell out quickly ■ Monitoring of pupil eating
Aspects of ‘healthy’ eating	■ Consumption of fruit, vegetables and salad ■ Strategies that would inspire pupils to eat healthier ■ Consumption, restriction and selling of ‘unhealthy’ food options ■ Drink consumption ■ Pupil perception of healthier eating
School food architecture intervention implementation	■ Pupils’ perception of dining room changes

**Table 2:** Themes and sub-themes from school staff interviews.

Theme	Sub-Themes
School food provision is important	■ Pupils have the opportunity of a daily hot meal ■ Pupils have access to healthy food ■ School food influences pupil’s eating behaviour
School catering practices	■ Development of menus ■ Complying with school food regulations ■ Responding to pupil requests and demands
Types of foods pupils buy	■ Snack foods ■ Fruit, vegetables and salads ■ Things that influence pupil choice
Day-to-day serving practices and issues	■ Food pricing and information ■ Serving food methods ■ Dining hall atmosphere ■ Queuing and time limits ■ School rules
Drinks and foods considered to be less healthy	■ Drink availability ■ The consumption of less healthy foods ■ The school has rules restricting ‘junk’ foods
Nutrition, health awareness and education	■ Pupil’s nutritional needs ■ Educating pupils about nutrition
Perceptions and knowledge of the school foods intervention	■ Staff understanding of the intervention ■ Views of the intervention ■ Impact of the intervention ■ Negative effects of the intervention
How school food provision could be improved or enhanced	■ School plans to improve school food provision ■ School foods improvement ‘wish-list’

## Appendix A. Focus Group Topic Guide

1. *Experience of taking school lunches*
  - Can you tell me about your experience of having a school lunch?
    ○ Timing
    ○ Atmosphere
    ○ Organisation
    ○ Payment method/system
  - What sort of food and drink do you normally select for your school lunch?
  - What influences your decision on what food and drink to choose?
    ○ Dining hall layout
    ○ Food availability
    ○ Card Vs cash
    ○ Other lunchtime commitments?
    ○ Any ‘unspoken rules’?
2. *Satisfaction of available foods*
  - What do you think about the food and drink choices available in your school?
  - Are there other types of foods/drinks/snacks you would like to be available?
    ○ Meal deals?
  - Are there any changes you would like to be made to the available food and drinks?
    ○ Why?
    ○ What?
    ○ Availability of water?
    ○ Cost of drinks?
3. *Knowledge and views of food and health*
  - (Can you tell me what you know about healthy eating?) Younger group only
    ○ What do you think are healthy food and drinks in school?
  - Do you think young people want to make healthier food and drink choices?
    ○ Why?
  - How do you think pupils can be helped to make healthier food and drink choices when buying food and drinks at school?
4. *Views of school food regulations*
  - Do you know of any rules in your school about the food and drinks on offer?
    ○ Any ‘unspoken rules’?
  - How would you feel if certain foods and drinks were banned in school?
    ○ What do you think about schools being able to restrict the types of foods available for you to buy?
  - Can you tell me about any changes you might have noticed in your school canteen recently?
    ○ Relating to how certain foods and drinks were promoted

Relating to how certain food and drinks were positioned

## Appendix B. Staff Interview Topic Guide

1. Importance of school food
  - Can you tell me your views of school food provision?
    ○ In relation to other school activities/priorities
    ○ In relation to pupil’s health
      ■ Whose role is it to promote health to pupils?
    ○ In relation to pupil’s behaviour
  - What do you feel about the restriction of certain types of foods and drinks in schools?
    ○ Is it feasible/acceptable to do so?
2. School practices
  - Can you describe how your school food provision operates?
    ○ Policies
    ○ Menus
      ■ Sugar awareness
      ■ Compliant drinks—what does this mean?
    ○ Food purchasing
    ○ Pupil payment system
    ○ Day-to-day practice
    ○ Kitchen/dining facilities
    ○ Pupil use/uptake
      ■ Pupil involvement in decision-making?
    ○ Pupil adherence to ‘rules’/practices
      ■ How is it organised/applied?
      ■ Who is responsible?
    ○ Monitoring of practices/processes
    ○ Staff involvement/practice in the dining room
3. School food intervention
  - Can you describe the food/drink intervention that recently took place in your school?
    ○ What changes were made?
    ○ Who was responsible for making the changes?
    ○ Were the changes noticed by anyone?
      ■ Who? Comments?
    ○ Were the changes practical/feasible?
      ■ Describe any difficulties/issues
      ■ Can you describe any unexpected findings?
  - Positive and negative?
    ○ How could the intervention have been done differently?
    ○ If you were able to make *any* changes you wanted to school food provision what would they be and why?
